# Orbital hydatid cyst and its successful treatment: A case report from Afghanistan

**DOI:** 10.1016/j.ajoc.2024.102140

**Published:** 2024-08-10

**Authors:** Sayed Farooq Hosaini, Abdul Qadir Qader, Mehrab Neyazi, Nosaibah Razaqi, Prakasini Satapathy, Habibah Afzali, Ahmad Neyazi

**Affiliations:** a Herat Medical Faculty, Herat University, Herat, Afghanistan; b Radiology Department of Herat Medical Faculty, Herat University, Herat, Afghanistan; c Afghanistan Center for Epidemiological Studies, Herat, Afghanistan; d Center for Global Health Research, Saveetha Medical College and Hospital, Saveetha Institute of Medical and Technical Sciences, Saveetha University, Chennai, India; e Scientific Affairs, Herat Regional Hospital, Herat, Afghanistan

**Keywords:** Hydatid cyst, Surgical management, Albendazole, Pediatric patient

## Abstract

**Purpose:**

This study aims to underscore the rarity of intraorbital hydatid disease caused by Echinococcus granulosus, emphasizing the importance of comprehensive exploration and documentation for effective management.

**Observations:**

Clinical presentations include proptosis, visual deterioration, ocular motility disruptions, and chemosis. A case study of an 8-year-old boy with a retroorbital hydatid cyst successfully resected through a right transcaruncular orbitotomy approach is presented, highlighting surgical complexities and the efficacy of pre and post-operative albendazole therapy.

**Conclusion and Importance:**

The successful excision and management of the intraorbital hydatid cyst underscore the significance of accurate diagnosis and precise surgical intervention. This case emphasizes the importance of expanding knowledge about this rare manifestation, contributing to enhanced diagnostic and treatment strategies for optimal outcomes in managing global health concerns.

## 1 Introduction

Cystic Hydatid disease, caused by the larval stage of Echinococcus granulosus,^1^ remains a significant global health concern, with reported cases spanning regions such as the Middle East, India, Africa, South America, New Zealand, Australia, Turkey, and Southern Europe.^2^ While this parasitic zoonosis has been extensively studied, intraorbital hydatid disease stands out as an exceptionally rare manifestation within the spectrum of hydatid cysts.^2^ This rarity underscores the importance of in-depth exploration and documentation of such cases to contribute to the body of medical knowledge. The clinical presentation of intraorbital hydatid disease encompasses a constellation of symptoms, including progressive proptosis accompanied by disruptions in ocular motility, visual deterioration, and chemosis.^3^ This unique array of symptoms further emphasizes the complexity and nuanced nature of this condition. In the realm of treatment, surgical intervention remains the cornerstone for managing hydatid cysts. However, retrobulbar lesions present a unique subset of complexities and risks. Complete removal of the cyst from this intricate anatomical location necessitates a skilled surgical approach, given the potential for cyst rupture and subsequent complications.^4^ Thus, the intricacies surrounding the surgical management of retroorbital hydatid cysts underscore the need for careful consideration and precise execution. In this report, we present a unique case of an inferiorly located orbit hydatid cyst, successfully excised through a right orbitotomy approach, with the patient experiencing an uneventful recovery.

## 2 Case report

**Fig. 1:**
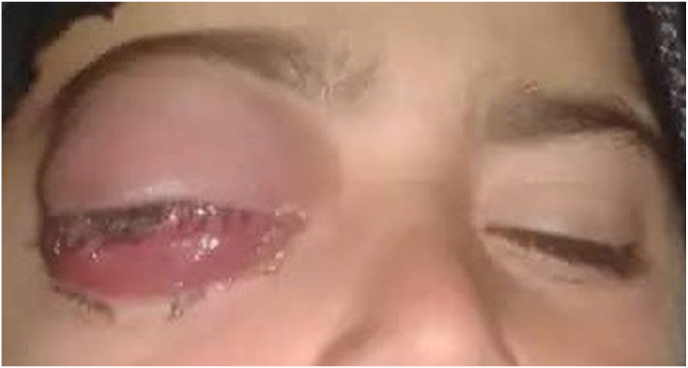
Preoperative images showing severe chemosis and proptosis of the right eye.

**Fig. 2:**
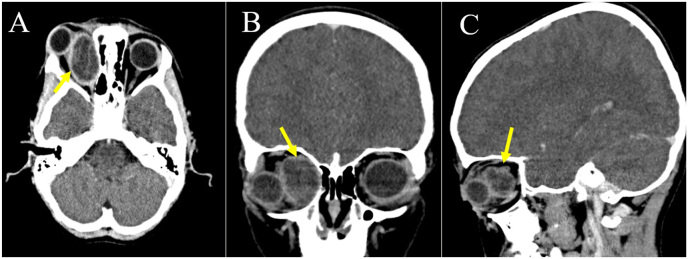
Axial (A), Coronal (B) and Sagittal (C) images showing well-delineated cyst (arrows) with septations located along the posteromedial wall of right orbit with lateral and upward displacement of the globe.

**Fig. 3:**
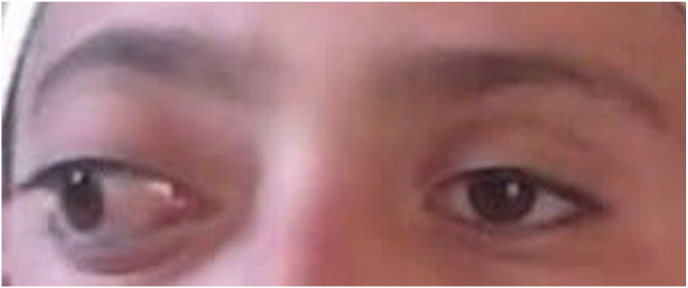
Preoperative images showing proptosis of the right eye.

**Fig. 4:**
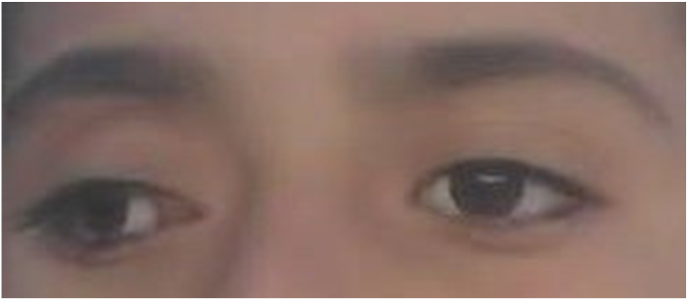
Postoperative images showing resolving of the chemosis and proptosis of the right eye (after twelve weeks of follow up).

In the presented case, an 8-year-old boy exhibited symptoms of eye swelling, chemosis, ocular discomfort, reduced vision, and headaches persisting for three months prior to seeking medical attention **[**Fig. 1**]**. On ophthalmological examination, normal anterior segment and clear media in the posterior segment. No afferent dysfunction or optic disc swelling/pallor was found on the dilated fundus exam. The eosinophil count was determined to be 0.3 × 10^3 cells per microliter (μL) ahead of the surgery. CT-Scan revealed posteromedial quadrant of the right orbit with well-delineated cyst with septation, along with lateral and upward displacement of the globe and caused outward bowing of the orbital structures with no associated bone destruction and the CT images revealed a medial right orbital cystic mass with rim enhancement and globe displacement **[**Fig. 2**]**. The patient received oral albendazole 400 mg once daily for 8 weeks pre operation. Following treatment symptoms of eye swelling and chemosis reduced, however proptosis still remained significant [Fig. 3]. The orbital hydatid cyst was surgically removed via orbitotomy. Due to its medial extraconal location within the orbital cavity, the surgery was conducted using a transcaruncular (medial transconjunctival) incision. Although the cyst was ruptured during the surgery but whole capsule and its contents were suctioned subsequently, the orbital cavity irrigated with normal saline prior to wound closure and wound closure made by 6/0 Vicryl sutures. Immediately after surgery Oral albendazole advised 400 mg once daily for 12 weeks. There was no clinical recurrence of proptosis after follow up imaging and normal vision 6/6 and postoperative showing resolving of the chemosis and proptosis of the right eye. Fig. 4 also shows right exotropia post-treatment, indicative of residual ophthalmoplegia **[**Fig. 4**]**

## 3 Discussion

Hydatid cysts of the orbit are rare and account for 1 % of all hydatid cysts.^5^ A survey of central nervous system hydatidosis cases in Turkey found that out of 336 cases, 22 were located in the intraorbital space.^6^ Typically, an orbital hydatid cyst is unilateral and occurs with or without hydatid cysts located elsewhere in the body.^6^ The most frequent clinical findings are exophthalmos, chemosis, lid edema, visual impairment, and restriction of extraocular motility.^7^ An orbital hydatid cyst tends to involve the retrobulbar tissues either within the muscle cone or outside in the superolateral or superomedial angle.^8^

Imaging modalities, particularly CT and MRI, play a pivotal role in characterizing cystic orbital lesions and narrowing down the differential diagnosis. CT scans offer detailed anatomical assessment and can delineate features such as calcifications within dermoid cysts or expansion of sinus walls in mucoceles. MRI provides superior soft tissue contrast, aiding in the differentiation of cystic lesions from surrounding structures and facilitating the identification of characteristic signal intensities. In the current case, the CT-scan findings revealed a well-delineated cyst with septation in the posteromedial quadrant of the right orbit, accompanied by lateral and upward displacement of the globe. The absence of associated bone destruction and outward bowing of orbital structures suggested a non-aggressive lesion. Additionally, the presence of fluid attenuation and mild rim enhancement within the cyst further narrowed the differential diagnosis.

The primary course of action for treatment involves complete surgical removal.^4^ There have been multiple surgical methods employed to access an orbital mass.^2^ It is crucial to have a grasp of the orbit's microanatomy and to select an appropriate surgical approach in order to minimize the risk of complications during the treatment of intraorbital hydatid cysts. Puncturing and irrigating hydatid cysts in the orbital cavity can minimize accidental rupture, control spillage, and preserve surrounding structures. However, it may not ensure complete cyst removal and poses risks of dissemination and limited applicability.^10^

Commencing albendazole treatment, particularly when initiated 14–28 days prior to surgery, serves as an adjunctive therapy alongside the surgical approach.^9^ Our administration of albendazole was aimed at reducing the risk of relapse. The resulting cosmetic outcome was highly satisfactory. Ultimately, early detection and timely surgical intervention for orbital hydatid cysts yield excellent outcomes for the majority of patients. This underscores the critical importance of accurate diagnosis. Clinicians should consistently consider hydatid cysts in the list of potential diagnoses for orbital masses, particularly in pediatric cases. The most significant surgical complication lies in cyst rupture during excision, which may lead to relapse. Nevertheless, achieving complete cyst removal without rupture is exceedingly challenging. In our case, we successfully accomplished complete extirpation; histological examination stands as the sole definitive confirmation.

## 4 Limitation

The patient underwent an extended pre-operative treatment course of 8 weeks due to limitations imposed by residing in a remote area, deviating from the optimal 14–28 days duration.

## 5 Conclusion

The present case enriches the understanding of intraorbital hydatid disease, providing clinicians with valuable insights into its clinical presentation, diagnostic considerations, and successful surgical management. Continued documentation of such cases contributes to the ongoing efforts to enhance the medical community's knowledge and informs future treatment strategies for this rare and challenging condition.

## Patient consent

The legal guardians of the patients provided both oral and written consent for the publication of the cases. This report has been carefully crafted to exclude any personal information that could potentially lead to the identification of the individuals involved.

## Sources of funding

None.

## Ethical approval

N/A.

## CRediT authorship contribution statement

**Sayed Farooq Hosaini:** Writing – review & editing, Writing – original draft, Conceptualization. **Abdul Qadir Qader:** Writing – review & editing, Writing – original draft, Conceptualization. **Mehrab Neyazi:** Writing – review & editing, Writing – original draft, Conceptualization. **Nosaibah Razaqi:** Writing – review & editing, Writing – original draft, Conceptualization. **Prakasini Satapathy:** Writing – review & editing. **Habibah Afzali:** Writing – review & editing, Writing – original draft, Conceptualization. **Ahmad Neyazi:** Writing – review & editing, Writing – original draft, Supervision, Conceptualization.

## Declaration of competing interest

The authors declare that they have no known competing financial interests or personal relationships that could have appeared to influence the work reported in this paper.
